# Linking ClinicalTrials.gov and PubMed to Track Results of Interventional Human Clinical Trials

**DOI:** 10.1371/journal.pone.0068409

**Published:** 2013-07-09

**Authors:** Vojtech Huser, James J. Cimino

**Affiliations:** Laboratory for Informatics Development, NIH Clinical Center, Bethesda, Maryland, United States of America; Children's Hospital of Eastern Ontario, Canada

## Abstract

**Objective:**

In an effort to understand how results of human clinical trials are made public, we analyze a large set of clinical trials registered at ClinicalTrials.gov, the world’s largest clinical trial registry.

**Materials and Methods:**

We considered two trial result artifacts: (1) existence of a trial result journal article that is formally linked to a registered trial or (2) the deposition of a trial’s basic summary results within the registry.

**Results:**

The study sample consisted of 8907 completed, interventional, phase 2-or-higher clinical trials that were completed in 2006-2009. The majority of trials (72.2%) had no structured trial-article link present. *A* *total* of 2367 trials (26.6%) deposited basic summary results within the registry. *Of* *those* , 969 trials (10.9%) were classified as trials with extended results and 1398 trials (15.7%) were classified as trials with only required basic results. The majority of the trials (54.8%) had no evidence of results, based on either linked result articles or basic summary results (silent trials), while a minimal number (9.2%) report results through both registry deposition and publication.

**Discussion:**

Our study analyzes the body of linked knowledge around clinical trials (which we refer to as the “trialome”). Our results show that most trials do not report results and, for those that do, there is minimal overlap in the types of reporting. We identify several mechanisms by which the linkages between trials and their published results can be increased.

**Conclusion:**

Our study shows that even when combining publications and registry results, and despite availability of several information channels, trial sponsors do not sufficiently meet the mandate to inform the public either via a linked result publication or basic results submission.

## Introduction

The purposes of trial registries, such as ClinicalTrials.gov, include serving as a repository for shared clinical trials results and providing the ability to track journal articles reporting trial results in medical literature. ClinicalTrials.gov registry has been documenting trials since 2000 and is the world’s largest registry. Of particular note, it provides electronic access to its database. It is an important tool for many constituents, including researchers (14%) and potential research volunteers (23%), with 95 million page views per month and 60,000 unique visitors daily [1,2].

We are interested in exploring mechanisms for automatically identifying results of completed trials that have either shared data sets within the registry or linked the trial records to published, peer-reviewed journal articles. In this study, we combined ClinicalTrials.gov data with data from PubMed, the popular citation database from the National Library of Medicine (NLM), to look at a large set of clinical trials and investigate how results of trials are made publicly available. We include in our study, an analysis of change in the number of trials reporting their results in some form since the establishment of policies and laws that encourage such reporting. Previous reports show that 32% to 53% of trials do not report their outcome [3,4]. To our knowledge, our study is the first study that combines publication data with deposited basic summary results. Our study also looks at the largest set of registered trials compared with previous publication rate analyses.

### Policies for Reporting Trial Results

From an evidence-based perspective, clinicians and researchers need to know about all human clinical trials in a given domain and consider results of all of them. Worldwide, there are policies of varying strengths that encourage publishing trial results in journal articles and legal mandates to report data about trials to clinical trial registries. In the US, principal investigators of trials supported by National Institute of Health (NIH) grants are encouraged to publish journal articles reporting trial results [5]. There are generally two types of mandates related to the use of trial registries: those related to *trial registration*, concerned with providing basic trial metadata, such as title, brief description and location to a trial registry, and those related to *trial result reporting*, concerned with extending the mere trial registration with submission of trial’s basic summary results. These two mandates (registration and reporting) are complemented by *medical journal policies*. The Uniform Requirements for Manuscripts (URM) policy created by the International Committee of Medical Journal Editors (ICMJE) has international scope and requires timely trial registration prior to enrolling the first trial participant, as well as proper linking of published journal articles to trial registration records. As a result of these mandates, prospective clinical trials registration has become a standard practice [6]. Within the US, a researcher typically starts a trial and, to facilitate recruitment and to comply with medical publisher’s policies [7], he or she registers the trial in a trial registry [8]. As the trial progresses, the trial principal investigator or trial record manager can later update this registration [9]. For example, he can change the trial status from “recruiting” to “closed to recruitment”, “completed” or “terminated”, and can attach references to journal articles reporting on the trial protocol or results. Since 2007, applicable trials covered by the Food and Drug Administration Amendments Act (FDAAA) [10] are required within one year from trial completion to update the trial registry record with basic summary results data (we use a shorter term *basic results*) in the form of a table of values for each primary and secondary outcome measures for each arm of the clinical trial [11]. Additional types or results (which we refer to as *extended results*, described below) may optionally be submitted. Trial results can thus be made publicly available via two result artifacts: by (1) publishing a journal article with trial results, or by (2) submitting basic summary results to trial registry.

### Links between Trials and Journal Articles

A *result article* is a journal article or monograph that reports some or all results of a clinical trial. Ideally, every trial should have at least one result article, even trials with non-significant or negative findings, although these may be harder to publish due to publication bias [12]. By publication bias we mean the fact that authors are more likely to submit, or editors accept, positive than negative (or inconclusive) results.

A trial can be linked to a journal article through an *unstructured trial-article link*, which is a link that does not involve unique identifiers. For example, (1) a given trial record in a repository may contain a free-text reference to a journal article that does not include a unique article ID (2), or a journal article mentions a trial name or acronym but does not unambiguously identify a registered trial. While potentially useful for readers of the article or registry record, such links to do not lend themselves well to automated retrieval and computerized analysis.

A trial can also be linked to a journal article through a *structured trial-article link*, which is a computable link that identifies both the trial and the journal article by a unique identifiers (e.g., the ClinicalTrials.gov ID “NCT00461032” and the PubMed ID “PubMed: 20674830”). We distinguish between two types of structured links based on their origin. An *abstract link* (or trial ID link) is a structured link created at the time of manuscript submission by the manuscript authors and facilitated by the journal’s editorial policies. Per ICMJE guidelines, when an article includes a trial ID in its abstract, that ID can be used to create an explicit, structured link that can be extracted through simple text processing of the article abstract. PubMed’s current indexing process automatically does so for two trial registries (ClinicalTrials.gov and ISRCTN). In the case of ClinicalTrials.gov, the trial ID is also referred to as an NCT number and the abstract link is sometimes referred to as an NCT link. A *registry link* (sometimes referred to as a PMID link) is a type of a structured link created by trial registry record manager and stored with the trial record in the registry. While some trial registries do not allow updates to trial metadata, ClinicalTrials.gov allows trial record managers to add result article references at any time after publication of a trial result article.

### PubMed

Several features of PubMed are useful for studies of trial result reporting. PubMed provides the ability to search for articles by a secondary ID (contained in the PubMed search field “[SI]”). This secondary identifier field contains accession numbers to various databases (e.g., molecular sequence data, gene expression or chemical compounds) that were detected in the indexed article. As per ICMJE’s guidelines since September 13, 2005, all clinical trial journal articles must clearly reference this trial registry ID in the article abstract when reporting trial outcomes. The NLM extracts those trial IDs and places then into the PubMed secondary ID field, creating computable abstract links as defined above. This greatly simplifies searching for result articles. For example, to search for journal articles resulting from trial NCT00000419, one can type the “NCT00000419 [SI]” into the PubMed user interface. Alternatively, one can use PubMed’s application protocol interface (API) called e-Utils, which is accessed through a Uniform Resource Locator (URL) such as “http://eutils.ncbi.nlm.nih.gov/entrez/eutils/esearch.fcgi?db=pubmed & term=NCT00000419 [si]”.

### ClinicalTrials.gov

The ClinicalTrials.gov website provides the ability to view individual trials metadata on the web, to search for trials by several basic parameters (e.g., by trial status: “Completed” or “Terminated”), to extract a tab-delimited file for up to 20 basic parameters, and to obtain full trial data in eXtensible Markup Language (XML) format. Individual XML files of selected trials can be obtained via a URL-based API. For example, the URL “http://clinicaltrials.gov/show/NCT01092702?resultsxml=true” can be used to obtain registration and results data for trial NCT01092702. The tab-delimited file and the XML file can be used to augment the website search to analyze parameters not accessible via the ClinicalTrials.gov website, such as “Primary Completion Date” in the tab-delimited file or “Number of Arms” in the XML file. Of note to our study is the fact that the Web view of a given trial includes PubMed article IDs for both abstract-linked and registry-linked articles; however, the XML file contains IDs for only registry-linked result articles. This limitation imposes a need for a separate query to PubMed to complement the XML trial metadata. Despite this small limitation, the XML-based format for downloading trial’s basic results is extremely flexible and can accommodate a wide range of trials. This flexibility is achieved by a schema that first defines the trial arms (e.g., “Intervention Arm” and “Control Arm”) and trial outcomes (e.g., “Symptom Free Days”), and later uses a generic tabular format for basic summary results as numerical values (participant counts or results of pre-defined outcome measures) for each defined trial arm and outcome.

## Methods

### Trial inclusion criteria

For this study, we considered clinical trials that met the following criteria:

• The trial is of interventional type and of phase 2 or higher (rationale: mandates for trial registration and basic results submission do not cover Phase 1 trials)
• The trial was first received by ClinicalTrials.gov between January 1^st^, 2006 and December 31^st^, 2012 (rationale: ICJME policy for mandatory registration came into effect September 13^th^, 2005)
• The trial has status “completed”
• The trial has valid start and primary completion dates
• Trial’s primary completion date is between January 1^st^,2006 and December 31^st^, 2009 (rationale: this ensures that the trials has had at least 3 years to publish a journal article with trial results or submit basic result to the trial registry)

### Trial metadata acquisition

Clinical trials for analysis were obtained by querying ClinicalTrials.gov on January 7^th^, 2013. For all trials that met the inclusion criteria, we obtained detailed trial XML data using ClinicalTrials.gov’s API, which provided registry trial-article links. The XML data also contained basic results for each trial where trial record managers had provided such results. We used the trial IDs to query the PubMed API on January 7^th^, 2013 to identify journal articles that referenced the trials via abstract links. The merged ClinicalTrials.gov data and PubMed data were analyzed for trial-article links and basic results data.

### Trial-article links

We separately characterized both types of structured trial-article links (abstract link vs. registry link). For each type, we looked at the number of trials having such links and the number of articles linked to a given trial. Because earlier studies [13] have indicated that most trials with linked articles have only a single article, we determined the proportion of trials that included single links and multiple links. We also analyzed trials that had both types of links present to determine when the links overlapped (that is, referenced the same articles) and when the registry link contributed new articles.

### Basic results data

We examined the ClinicalTrials.gov data to identify the presence of trial results. Trial results are submitted by trial record managers on behalf of the trial sponsor and principal investigator. The ClinicalTrials.gov registry does not store individual patient result data but only accepts results aggregated per study arm, such as count of patients or numerical measures (e.g., “percentage of arm patients that are symptom free at the end of the study”). Trial results are organized into four modules (as defined by the FDAAA): (1) participant flow, (2) baseline characteristics, (3) outcome measures, and (4) adverse events. Within each module, there are required items and optional items [14]. For baseline and outcome measures, the results could be a number (e.g., number of participants), or descriptive statistics with a central tendency value (e.g., mean, median, or geometric mean) and a dispersion value (e.g., standard deviation). Both categorical, as well as continuous measures can be entered and are required to be reported separately for each study arm or compared group. No effort was made to classify clinical trials as trials with positive or negative overall findings either from basic results or linked articles and thus we did not investigate such questions as whether negative trials are less likely to publish related articles.

To facilitate presentation of our results, we define two types of basic results:

• Trials with *required results* are trials that only provided answers to the legally required basic summary result items, such as number of participants starting and completing each trial arm, gender and age baseline characteristics, at least one outcome measure and a table of adverse events. An alternative term for this class of trials would be “trials with only legally required basic summary results” or “trials with minimum necessary basic summary results”.
• Trials with *extended results* are trials that, in addition to providing the legally required basic summary result items, also provided optional result items. These were guided by standards for Good Clinical Practice and CONSORT statement and referred to as “Good reporting practice” [15]. Optional items in participant flow report additional counts of patients “Lost to follow-up” or “Severe Non-Compliance to Protocol”. Most importantly, optional items in the outcomes measures module provide p-values of statistical significance of each individual primary or secondary outcome measure and information about the statistical tests used. In our analysis, we considered the presence of at least one statistical analysis method (and associated p-value) as sufficient for classification as extended results. This parameter was chosen because it enables most directly to assess the overall outcome of the trial into positive and negative trial and potentially later explore the questions such as publication bias.

### Overall results availability over time

Finally, we noted the frequency with which trial results are available via the registry in any form by combining the availability of basic results with the presence of linked result articles, with a subgroup analysis based on the year of trial completion. We also examined the conditional dependence between the presence of linked article and provision of basic results.

## Results

### Sample description

In total, 138,153 trials were registered in ClinicalTrials.gov as of January 7^th^, 2013. Of these, 113,112 were received between January 1, 2006 and December 31, 2012; 47,067 trials had “Completed” status, of which 21,961 trials were interventional studies of phase 2 or higher. When studies were excluded based on start and primary completion date criteria, 8907 eligible trials remained for evaluation.

### Trial-article links

A total of 2068 (23.2%) trials had one or more abstract trial-article links present. For trials with abstract links, there were 1.24 articles per trial with 84.4% trials having a single linked article. A total of 647 (7.3%) trials had one or more registry links. For trials with registry links, there were 1.62 articles per trial, with 78.1% of trials having a single linked article. Without regard to the trial-article link type, a total of 2477 (27.8%) trials had at least one linked article, with 1.46 articles per trial on average and 76.4% of trials having a single linked article. The majority of trials (6430/8907; 72.2%) had no structured trial-article link present.

Only 238 trials (2.7%) had both types of links. From the results above, it is clear that the abstract links are much more prevalent than the registry links. In our analysis of the links overlap within those 238 trials, we hence focused on the question of how often the less prevalent registry link adds new articles that are not covered by the more prevalent abstract link. We found that in 92 trials (1.03% out of 8907) the registry link added new linked articles not covered by the abstract link.

### Trials with basic results

**Figure 1:**
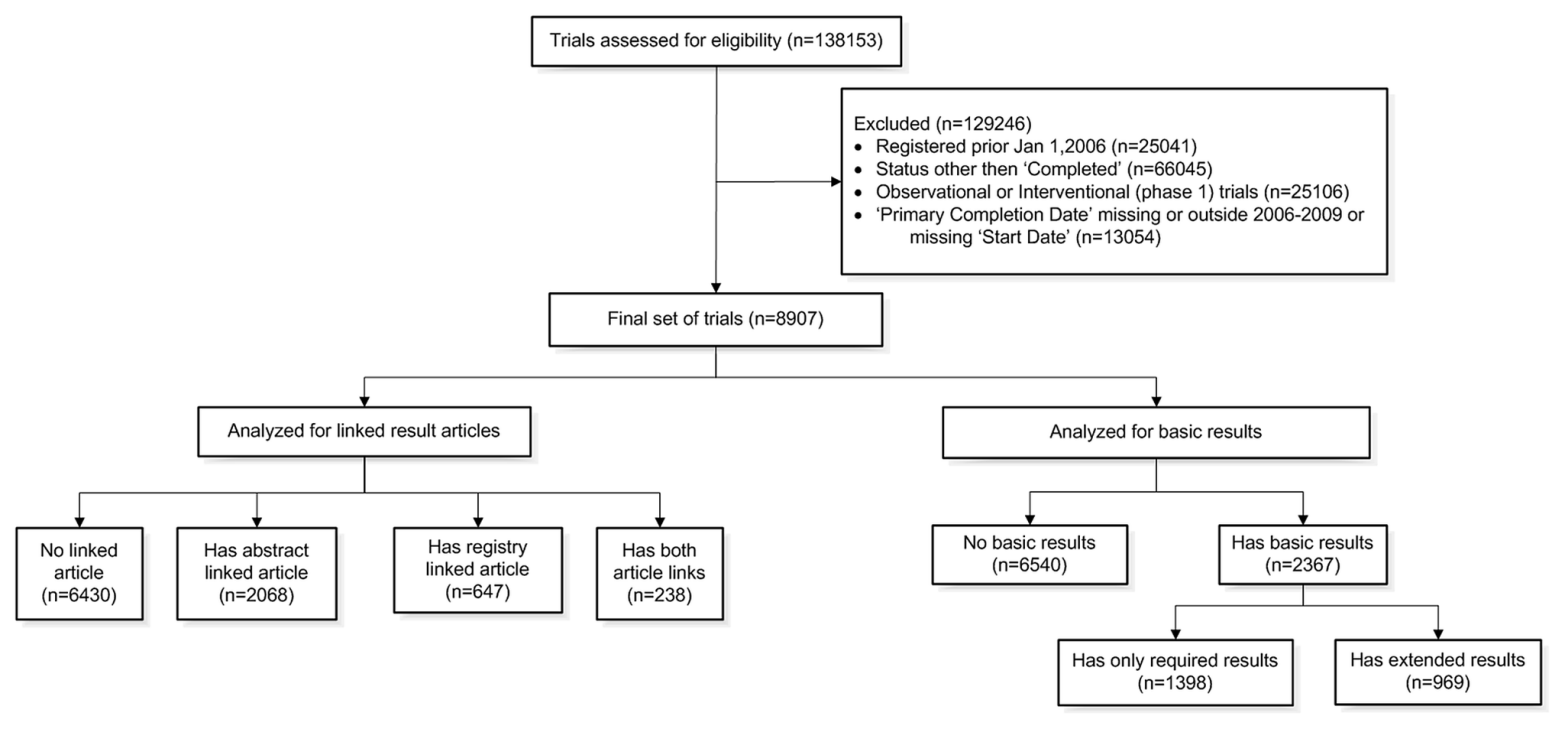
Trial selection and analysis for presence of linked result article and basic results.

A total of 2367 trials (26.6%) provided basic results. Of those, 969 trials were classified as having extended results (providing statistical method and p value for at least one trial outcome), representing 10.9% of all trials and 40.9% of trials providing results. The remaining 1398 trials (15.7% of all trials; 59.1% of trials providing results) were classified as trials with required results. Figure 1 provides an overview of trial groups analyzed, as well as the trial selection process.

### Association between presence of linked result article and trial basic results

**Figure 2:**
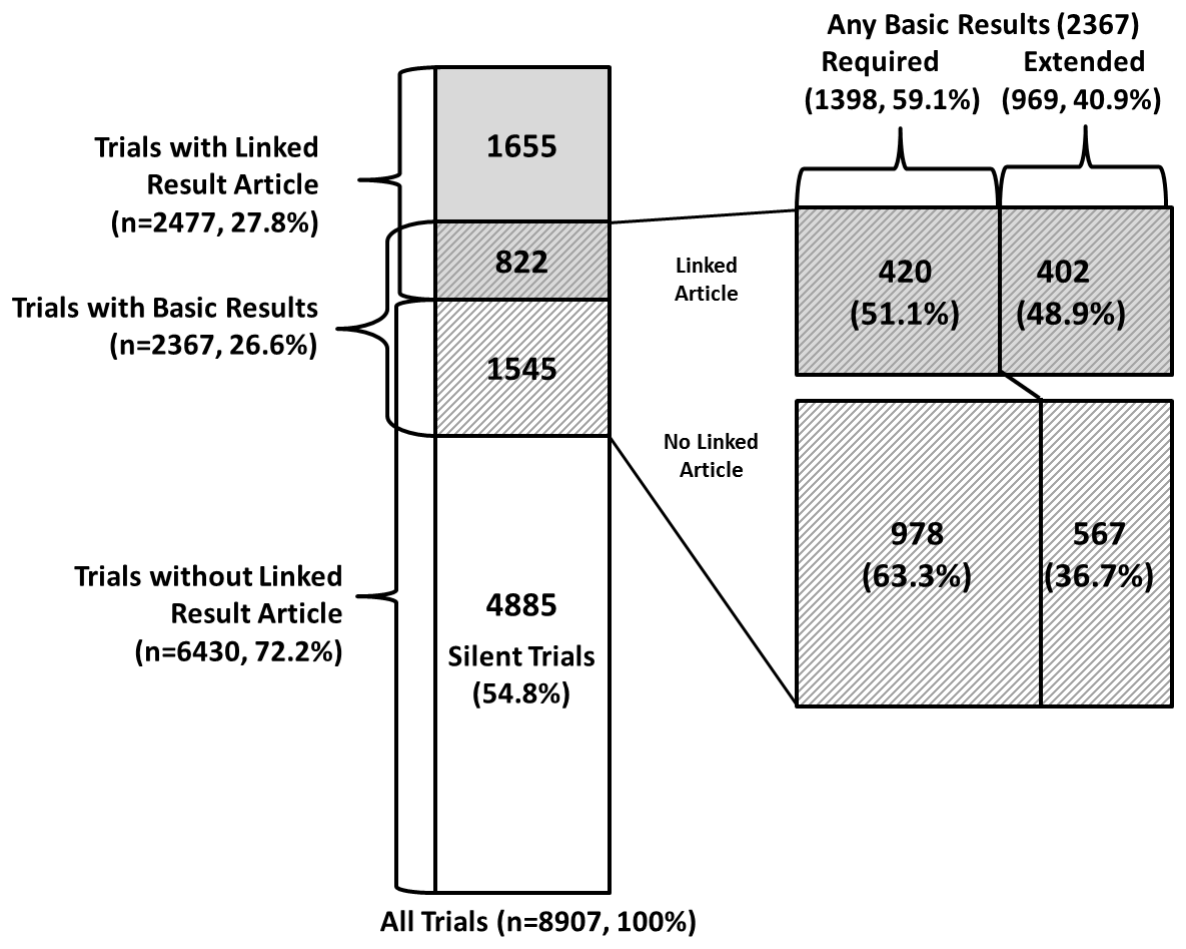
Diagram showing proportionally the overlap of trials with linked result article and trials with basic results and large proportion of silent trials with no linked result artifact. Enlargement to the right illustrates the disproportionate publication rate (linked result article) between studies that have extended results, compared to those with only required results.

Figure 2 shows the overlap of trials with linked articles and trials with basic results. For the purposes of considering the interdependence of these two types of result reporting, we considered registry-linked and abstract-linked articles together. Table 1 shows the numbers of trials with any linked article and trials with basic results; Table 2 shows the conditional probabilities between the various forms of result reporting.

**Table 1:** 2x2 table showing trials with linked articles and trials with basic summary results (available via the registry); trial counts are shown with percentage ratio in brackets.

	**Trials with Basic Results**	**Trials without Basic Results**	**Total**
**Trials with Linked Result Article**	822 (9.2)	1655 (18.6)	2477 (27.8)
**Trials without Linked Result Article**	1545 (17.3)	4885 (54.8)	6430 (72.2)
**Total**	2367 (26.6)	6540 (73.4)	8907 (100)

**Table 2:** Conditional probabilities showing relationship between presence of linked result article, required results and extended results.

**Trial Property (A)**	**P(A) (%)**	**Conditional probabilities**
Has linked result article	27.8% (2477/8907)	P(has linked result article | has required results) = 34.7% (822/2367)
		P(has linked result article | has extended results) = 41.5% (402/969)
Has no linked result article	72.2% (6430/8907)	P(no linked result article | has required results) = 65.3% (1545/2367)
		P(no linked result article | has extended results) = 58.5% (567/969)
Has required results	26.6% (2367/8907)	P(has required results | has linked result article) = 33.2% (822/2477)
		P(has required results | no linked result article) = 24.0% (1545/6430)
Has extended results	10.9% (969/8907)	P(has extended results | has linked result article) = 16.2% (402/2477)
		P(has extended results | no linked result article) = 8.8% (567/6430

From Table 1, we can observe that there are 4885 trials (out of 8907; 54.8%) that have neither a linked result article nor basic results submitted. We refer to such trials as *silent trials*, since there is no detectable result artifact for them. A total of 822 (9.2% out of 8907) trials provided both a linked result article and basic results. From the opposite point of view, there are 4022 trials (45.2% out of 8907) that have some indications of results (linked result article or basic results). For 1545 trials (17.3% out of 8907), the only indication of trial outcomes is the registry’s basic results submission.

From Table 2, we can observe that trials with required results have a 28% higher chance of having a linked result publication: general rate of 27.8% increases to 34.7%. The difference is even greater for trials with extended results. The inverse relationship is also true: trials with a linked publication have a 25% higher chance of having basic results submitted: (general rate of a 26.6% increases to 33.2%). The relationship between the presence of a linked result article and the type of basic results is also shown graphically in Figure 2 (enlargement part): trials with extended results are more likely to have a linked result article.

### Overall results availability over time

Table 3 presents various trial result parameters over a period of four years (2006-2009) that can be analyzed in our sample, showing the existence of abstract-linked articles, registry-linked articles, basic results and combined results availability. The trial’s primary completion date was used to temporally organize trials into four groups with the second column indicating trial counts. Basic results submission has increased over time from 10% (trials completed in 2006) to 30% (trials completed in 2009). Existence of abstract-linked article remained in the range of 23-24% and, similarly, existence of registry-linked article remained in the range of 6% to 8%. Availability of any result artifact (result article or basic results) increased from 35% (trials completed in 2006) to 47% (trials completed in 2009).

**Table 3:** Availability of trial results artifacts arranged by trial completion year.

**Trial Completion Year**	**All Trials in the Analyzed Sample**	**Trials with Some Results (Basic Results or Linked Article)**	**Trials with Basic results**	**Trials with Abstract Linked Article**	**Trials with Registry Linked Article**
2006	645	227 (35.2)	67 (10.4)	150 (23.3)	53 (8.2)
2007	1645	639 (38.8)	292 (17.8)	384 (23.3)	137 (8.3)
2008	3150	1531 (48.6)	982 (31.2)	755 (24.0)	235 (7.5)
2009	3467	1625 (46.9)	1026 (29.6)	779 (22.5)	222 (6.4)

## Discussion

Our study analyzes the body of linked knowledge around clinical trials (which we refer to as the “trialome” [16]) in terms of existence of linked result article or presence of basic summary results using data from the world’s largest biomedical literature citation database and the world’s largest clinical trial registry (ClinicalTrials.gov) [16]. The most important findings of our study are the following facts: 27.8% of trials provide linked result article; 26.6% provide basic results; the two ways of providing trial results overlap in 9.2% of trials; and 54.8% of trials are silent, when only formally linked results are considered.

Our analysis of the linked result articles shows that the majority of trials with linked articles (76%) have exactly one linked publication. In terms of the two types of structured trial-publication links, the abstract link is much more frequent than the registry link (2068 vs. 647). In the uncommon case of multiple linked publications (via any link type), the registry link provides more linked articles (mean 1.62 registry links vs. 1.24 abstract links); however, our prior study [13] showed that the abstract link is more precise, perhaps because it is subject to journal editor and peer review. With respect to automated analysis and use of the trialome, the abstract link is the more optimal way of linking trials to result articles. However, our results in Table 2 indicate that the two types of links overlap in only 2.7%, and that the registry link did contribute an additional 4.6% of trials that would otherwise have no linked result article. Therefore, providing trial record managers with the ability to submit a registry-linked result article (at any time and independently from the journal publication process) does provide some value. The registry link allows for retrospective linking of relevant result articles that did not include the trial ID in the original article abstract. In fact, the registry link feature could be used to increase the overall linkage rate of 28% of trials having a linked result article. Currently, only three out of the five largest trial registries allow submission of such links.

In terms of overall availability of linked result articles, our results show that a small number (less than 28%) of registered trials have such article links within 3 years after trial completion. This rate is lower than estimates of published trials rate found in studies that included unlinked result articles (46% [3] and 68% [4]). This discrepancy points to journals potentially allowing publication of clinical trial result articles without properly linking them to a registry trial ID. This is despite the URM policy adopted by more than 1000 journals requiring such linkage. Our earlier study of five high-impact journals showed 96% compliance with inclusion of trial registration ID within abstracts of articles of type “clinical trial” [16] and shows that successful URM enforcement is possible.

In terms of the second form of trial result reporting, basic results submission, our analysis points to an increasing trend of trial record managers making these submissions to the registry (increase from 10% in 2006 to 30% in 2009; Table 3). For a substantial portion of trials (1545/8907, 17.3%), submitted basic results are the only result artifact available. This percentage speaks to the significance of ClinicalTrials.gov results database.

The analysis of required vs. extended results shows that the majority of trials with results (59%) do not include any statistical analytical method and associated p-values, which makes interpretation of the findings as positive or negative difficult. Without such results, the interpretation requires extensive analysis of the study arm labels, the declared outcome measures and the tabular format presenting those measures across all study arms.

Our original study design included classification of trials into trials with overall positive and negative findings and a direct investigation of publication bias. However, our analysis of deposited basic results showed that such positive or negative overall trial classification cannot be easily performed computationally and requires extensive manual review by at least two experts. Overall trial classification is also made more complex by the increased use of non-inferiority testing methods and lack of necessary details in the result article abstract. Our study provides automated methods for collecting the data needed to answer the question “Is there a publication bias towards studies with positive results”, but analysis of those data will require significant manual, expert review with careful attention to inter-rater reliability.

Finally we want to highlight that our study shows a positive value of depositing basic results into the registry in terms of overall result availability. In our temporal analysis we observed that between 2006 and 2009 there was an overall increase in availability of any result artifact (linked result article or basic results) from 35% to 47%. However, Table 3 shows that this increase was almost entirely due to an increase in basic results submission (increase from 10% in 2006 to 30% in 2009), whereas the existence of linked result articles remained largely the same over time in the range of 6-8% for registry linked articles and 23-24% for abstract linked articles.

### Limitations

Our reported rate of 28% of trials having result articles only includes articles formally linked via the trial-article abstract or registry. A prior study of negative predictive value of trial-article links [13] shows that 44% of trials with no linked result articles indeed have an *unlinked result article* that can be found by manually searching PubMed. This reliance on formal structured trial-article links leads to underestimating the percentage of trials with result article but is consistent with our focus on informatics methods and structured trialome exploration. The missing links, however, can be retrospectively added using the registry link mechanism. Incentivizing trial sponsors and trial record managers to do so could eliminate this problem of unlinked result articles. The problem would also not exist, if the URM policy requiring trial ID in the article abstract would be followed by all journals.

In addition to omissions by manuscript authors, there is also a small risk of imperfect extraction of abstract trial-article links during PubMed indexing process. In our prior study analyzing 698 articles of publication type “Clinical Trial” [17], we observed that, in 2% of the articles, the PubMed indexing process did not properly populate the SI field with a trial ID clearly present in the publication abstract. This PubMed indexing limitation is outside of our control and could not be remedied by manual review due to PubMed size. The link extraction limitation has much smaller impact compared with the above issue of complete absence of trial-article link (2% vs. 44%).

Our study only considered publications available via PubMed and indexed in MEDLINE. Trial results can also be presented at conferences or in publications not available via PubMed. However, we think that readers of the medical literature will be likely to limit their search to PubMed.

By restricting our sample to trials completed prior Dec 31, 2009, our study only allowed a minimum of three years for result article to be published. By searching PubMed and ClinicalTrials.gov on January 7^th^, 2013, result articles or basic results that appeared after that date were not included in our analysis. However, prior studies indicate that the mean time to publish is 2.04 years and that 81% of studies that eventually publish and link a result article do so within 3 years after completion date [3,4,6,18].

Our study used primary completion date as the completion time of the study in the temporal trends analysis. ClinicalTrials.gov defines two types of study completion dates. The primary completion date is defined as the “last visit, last patient date” and that follows the definition specified in US Public Law 110-85, Title VIII, Section 801, with respect to an “applicable clinical trial”. The formal definition is “the date that the final subject was examined or received an intervention for the purposes of final collection of data for the primary outcome”. The second completion date type is called simply “study completion date” and defined as the “final date on which data were collected”. This second type of completion date technically allows for additional time in obtaining all study data and could possibly be some time after the primary completion date dictated by law. However, using the second completion type instead of the date specified by FDAAA would likely have only a small impact on results of the temporal trends in result artifacts since it delays trial completion by only 3.3 months on average.

### Comparison with other studies

Our study is unique in looking at the trialome from computational and informatics perspectives and in seeking multiple trial result artifacts by cross tabulating the existence of linked result articles with the existence of basic results.

There have been several prior studies that examined the rate of trial publication. Three studies were limited by investigating only a single clinical domain [19-21] or a single country [22] but the remaining four studies used sufficiently general trial inclusion criteria to be discussed in detail.

Two of these studies partially addressed the limitation introduced by unlinked result articles and included manual efforts to seek such articles. Ross and colleagues investigated 677 randomly selected interventional trials of phase 2 or higher completed prior to August 6, 2007 [3]. They determined the publication status through references within ClinicalTrials.gov and by manual efforts (searching PubMed using the trial keywords, and emailing the trial officials). In their dataset, 96 trials (96/677; 14%) had a linked result publication, which was adjusted upward to 46% (311 of 677) after manual search and email inquiry efforts. A second study by Ross et al., looked at a restricted sample of 635 NIH-funded trials that completed prior to December 31, 2007 [4]. This second study used a similar methodology to determine publication status (omitting, however, email to study officials). The overall publication rate was 68% (432 of 365 trials), with a time-restricted publication rate (within 2.5 years after completion) of 46%. The relative contribution of publications detected through links versus manual searching was not reported.

The third study by Zarin and colleagues investigated a restricted sample of 2324 trials (those that submitted study results to ClinicalTrials.gov) and reported that 14% of trials had a result publication obtainable by structured links. In a more detailed review of 150 randomly selected trials, this rate increased to 52% when manual PubMed searches were included [6].

The fourth study relied solely on automated methods. Kirillova et al. examined trial sponsor and trial design to identify factors that predict the deposition of basic results; they also reported a publication rate (9.5%) based solely on the registry trial-article links in ClinicalTrials.gov XML data. Unlike our study, their publication rate analysis did not include data available through the ClinicalTrials.gov web trial view or by combining the registry data with PubMed data [23].

Overall, these studies show linked-article publication rate ranging between 9% to 14% and a total publication rate of 46% to 68% (when manually retrieved articles are considered). Our study, in comparison, investigates a much larger trial sample and combines the analysis of the publication rate with the analysis of the availability of basic results. Our study is the first to comprehensively describe and investigate the relationship (overlap) of the two structured links: abstract link vs. registry link. It is also the first study to analyze the interdependence between publication linkage and basic result availability.

### Implications

Our results have several implications for the clinical research enterprise conducting human interventional trials. Some of these suggest possible changes of clinical trial registries while others are relevant to trial sponsors or legislators. Optimally, evidence-based medicine would be informed about all completed trials (*trial transparency*) and all trials would publish their results (*result transparency*). Accepting for the moment that clinical trial registries provide complete trial transparency, our investigation shows significant gaps in result transparency with over half of all trials (54.8%) being *silent* (no linked result article or basic results). We can imagine many possible reasons for this high proportion of silent trials (e.g., missing formal link to an unlinked result article; commercial interest in delaying results; technical data collection flaws; or trials with hard-to-publish negative scientific results). Of note, our inclusion criteria required trials to be formally declared as “completed” and we excluded trials terminated due to reasons of ethical problems or under-recruitment.

Trial sponsors might be more inclined to reduce their percentage of silent trials if their compliance rates were publicized (as in our File S1 that ranks trial sponsors within our sample by percentage of silent trails). The percentage of silent trials might also be reduced through legislative changes, perhaps included in the final rule-making process around FDAAA due in 2013, or through additional capabilities in ClinicalTrials.gov. For example, ClinicalTrials.gov might enable principal investigators to share result articles that were rejected by the journal due to insignificant results (“upload rejected result article manuscript”), and thereby mitigate publication bias; ClinicalTrials.gov might also accept narrative summaries of trial results. In the past, the option for allowing narrative summaries created by trial sponsors was criticized as potentially biased (not peer reviewed, created by trial sponsor). However, allowing it for only currently silent trials that lack any result artifact can provide at least some public result disclosure and limit individual public inquiries to study sponsor. Finally, ClinicalTrials.gov might allow trial sponsors to submit Web links to non-ClinicalTrials.gov trial summary data repositories (e.g., link such as PfizerTrialResultsMadePublic.com/trial/NCT00004200).

On the issue of FDAAA jurisdiction over trials in our sample, we briefly analyzed how jurisdiction over a given trail can be determined. ClinicalTrials.gov’s public XML schema contains a field ”is_fda_regulated” available under <clinical_study/is_fda_regulated> . This field is self-declared by trial record managers and may be incorrect. Using this field, 4292 trials in our sample (48.2% out of 8907) indicated they were FDA regulated. To determine trials’s FDA jurisdiction status, an important parameter would be to know whether a particular trial with a certain NCT ID is linked to an FDA Investigational New Drug (IND) or Investigational Device Exemption (IDE) application. This link to an IND or IDE number is known to ClinicalTrials.gov internally; however, for commercial interest protection reasons, it not exposed in the public XML file.

Our study of required versus extended results reporting relates to the question of whether current policies and mechanisms for basic summary results indeed contribute to full result transparency [24] and for what target audience such full transparency is directly desired. According to the 2007 survey of users of ClinicalTrials.gov website, patients and patient relatives represent 51% of ClinicalTrials.gov visitors [2]. Individual healthcare consumers increasingly research their own or family healthcare needs online and may lack the necessary expertise to interpret the tabular basic summary results. Moreover, even for a clinical or statistical expert, results that include the employed statistical tests and p-values are easier to interpret than raw summary tabular data. Yet, in our sample, 59% of results did not include statistical tests and p-values, making result interpretation difficult even for an expert. Scientific integrity would imply that all trials would report their results in a journal publication. In our view, reporting only basic summary results in tabular format with no peer-reviewed commentary has significant limitations and strengthening the medical literature reporting of trials, regardless of the overall outcome should not be dismissed by depositing the basic summary results. The fact that the ClinicalTrials.gov administrators provide the extended results reporting at least as an option for trial record managers, is a step in the right direction, but it is not mandated by the legislature and, as our results show, not used by the majority of basic results depositors.

## Conclusion

Within the field of clinical research informatics that strives to improve human clinical research in general, understanding and improving the informatics infrastructure of the trialome (a body of linked knowledge around human clinical trials) is of significant value. Our study presents an enhanced, automated method for viewing the current state of the trialome using the world’s largest trial registry and the world’s largest biomedical citation database. Only 47% of trials completed in 2009 analyzed in our sample provided linked publications or basic results. Despite availability of several information channels, trial record managers do not sufficiently meet the mandate to inform the public about results of clinical trials either via a linked result publication or basic results submission, although there is a temporal trend showing an increasing rate of submission of basic results.

## Supporting Information

File S1 Multi-tab spreadsheet file that ranks trial sponsors (of 8907 trials within our sample) by percentage of silent trials. Four tabs within the file list: (1) bottom 10 trials sponsors with lowest percentage of silent trials; (2) top 10 trial sponsors with highest percentage of silent trials; (3) silent trial percentages for 83 trial sponsors with 15+ trials in our sample; and (4) silent trial percentages for all 2285 trial sponsors within our sample.
(XLS)
